# Hypoxia and oxidative stress related to inflammation and vascular aspects of the pathogenesis of psoriasis

**DOI:** 10.1093/skinhd/vzaf007

**Published:** 2025-12-18

**Authors:** Sabina C A Hanssen, Marieke M B Seyger, Piet E J van Erp, Catharina J M van der Vleuten, Peter C M van de Kerkhof

**Affiliations:** Department of Dermatology, Radboud University Medical Center, Nijmegen, The Netherlands; Department of Dermatology, Radboud University Medical Center, Nijmegen, The Netherlands; Department of Dermatology, Radboud University Medical Center, Nijmegen, The Netherlands; Department of Dermatology, Radboud University Medical Center, Nijmegen, The Netherlands; Department of Dermatology, Radboud University Medical Center, Nijmegen, The Netherlands

## Abstract

Psoriasis is a systemic autoimmune disease with roles in the innate and adaptive immune systems. Histological features include aberrant vascularization with dilated, tortuous, thin-walled capillaries and a mixed inflammatory infiltrate with mononuclear cells and neutrophils. There is increasing evidence that oxidative stress (hypoxia) plays an important role in vascular and inflammatory processes in the pathogenesis of psoriasis. In addition, it appears that systemic inflammation and oxidative stress could be a mechanistic link between psoriasis and concomitant cardiometabolic disorders. To present a unifying overview of the current literature on the general concept on the interrelationship between oxidative stress, vascular alternations and inflammation within the pathogenesis of psoriasis. More particularly, we aimed to gain insight into the pathomechanisms related to cardiovascular comorbidities – an important and distressing component of psoriatic disease. Standardized literature searches in PubMed and Embase were carried out with a focus on oxidative stress, inflammation and vascularization in psoriasis. In this article, the current knowledge on the role of oxidative stress in the inflammatory and vascular aspects of the pathogenesis of psoriasis are stated. Moreover, contemporary awareness of the pathomechanisms related to cardiovascular diseases are pointed out. The review presents arguments to underline the importance of hypoxia and oxidative stress in the inflammatory and vascular response within the pathogenesis of psoriasis and associated various cardiovascular and metabolic diseases.

## Teaser

This review presents arguments to underline the importance of hypoxia and oxidative stress in the inflammatory and vascular response within the pathogenesis of psoriasis and associated vascular and metabolic diseases.

Psoriasis is a chronic T-cell-mediated systemic disease of inflammation affecting approximately 1–3% of people worldwide.^1^ Psoriasis involves the skin, joints, eyes, and cardiovascular and central nervous systems.^1–11^ With respect to the skin, psoriasis is characterized macroscopically by erythema, induration and scaling, reflecting inflammation with involvement of the endothelium, epidermal proliferation and abnormal epidermal differentiation, respectively. There are genetic factors that predispose for psoriasis and triggering factors may aggravate the disease.^1,12^ Psoriasis is a disease of systemic inflammation with involvement of innate and adaptive immunity.

Hypoxia modifies the activity of the cytochrome chain responsible for mitochondrial oxidative phosphorylation, resulting in a decrease in adenosine triphosphate (ATP) synthesis and increased reactive oxygen species (ROS) at the same time as a decrease in the activity of the cellular antioxidant system, which may lead to oxidative stress. Oxidative stress, defined as a disturbance in the balance in the production of ROS (free radicals and antioxidant defences), plays a pivotal role in cutaneous inflammation.^13,14^ Some research has already been done on oxidative stress in psoriasis. However, our insights are fragmental and the role of this process related to inflammation and vascular involvement has not been integrated in a unifying concept.

In recent years, there has been increasing interest in oxidative stress in the pathogenesis of psoriasis and a growing number of studies focus on trying to elucidate the interrelationship between oxidative stress and inflammation and/or vascular modification within this disease. Therefore, in this literature review, we aim to present a unifying overview of current findings and to present a general concept on hypoxia related to inflammation and vascular aspects in the pathogenesis of psoriasis.

In the past, psoriasis has been regarded as an autoimmune disease affecting only skin and joints. The current view is that psoriasis is a systemic disease of inflammation, associated with various comorbidities, including elevated prevalence rates of cardiovascular risk factors, such as hypertension, diabetes, obesity, dyslipidaemia and (subclinical) atherosclerosis, and an increased risk for developing severe vascular events, such as cardiovascular and cerebrovascular events.^1,10,11,15^

Numerous recent studies have unequivocally revealed that psoriasis is a systemic inflammatory disease, and many of the key drivers of psoriasis are also implicated in the pathogenesis of other common chronic inflammatory comorbidities. It appears that systemic inflammation and oxidative stress may be a mechanistic link between psoriasis and cardiometabolic disorders.^14^ Insights into these relationships could significantly help ameliorate primary and secondary prevention of the disease and obviate complications. Furthermore, psoriasis is associated with more comorbidities like depression and sleep disorders, probably as a manifestation of neuroinflammation.^2–5^

The aim of the present study was to review the current knowledge on the interrelationship between oxidative stress, vascular alterations and inflammation in psoriasis. More particularly, we aimed to gain insight into the pathomechanisms related to cardiovascular disease, an important and distressing component of psoriatic ­disease.

## Materials and methods

**Figure 1:**
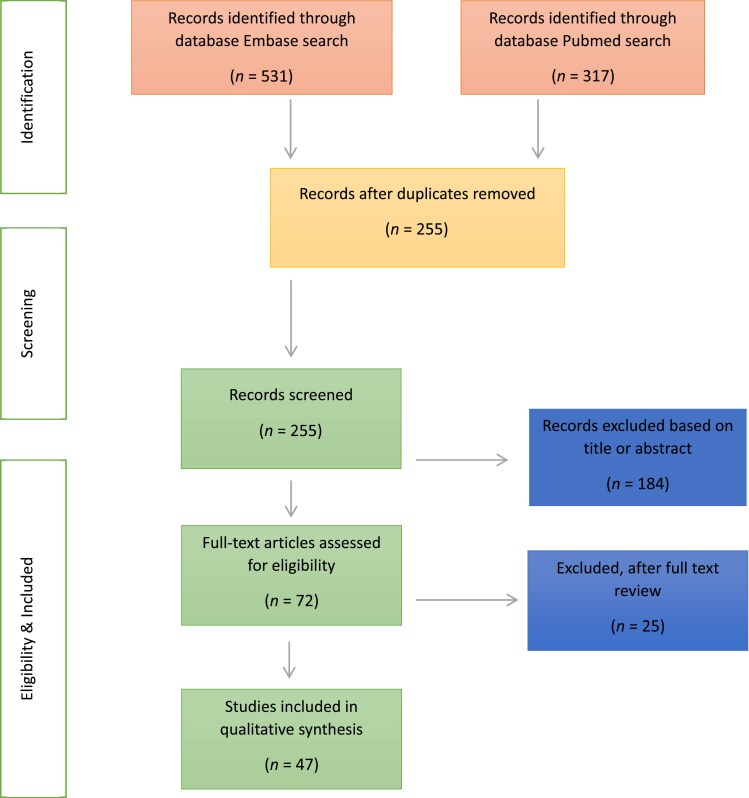
PRISMA flow diagram.

This literature review was performed by searching two electronic databases, PubMed and Embase, with a focus on articles containing the keywords ‘psoriasis’ in combination with oxidative stress (+ synonyms: hypoxia, cell hypoxia, hypoxia-inducible factor 1, oxidation-reduction, oxygen-defic*, anoxia, anoxem*, HIF1*, HIF-1* and oxidative stress*) and inflammation (+ synonyms: inflammation mediators, inflamm*, chemokin*, cytokine*, interleukin*, immunity*) and/or vasculature [+ synonyms: endothelium, neovascularization (physiologic and pathologic), vascular remodeling, vascular endothelial growth factors, blood vessel, endothelial cells, vascul*, neovasc*, immunovasc*, VEGF* and angioge*]. The results of these searches were subsequently selected based on a preselected examination method; the predetermined experiments included were RNA research projects, Western blot techniques, *in situ* hybridization and immunohistochemistry (keywords: RNA messenger, mRNA, RNA messeng*, Blotting – Western, Blott*, Western blot*, *in situ* hybridization, *in situ* hybridi*, immunohistochemistry, immunohisto*). The literature search was restricted to human studies and written in Dutch, English or German. The initial search resulted in 531 articles; after removal of duplicates 255 articles remained. All titles and, if necessary, abstracts were assessed by two separate reviewers (S.C.A.H. and P.C.M.K.) for usefulness on answering the devised research question. Differences in the assessment of an article were discussed by the two reviewers and resolved by consensus. Existing review articles and solitary animal studies were excluded. This method eventually yielded 47 articles (Figure 1); these 47 articles were predominantly used in the first two sections below discussing ‘Oxidative stress and vascular alterations in psoriasis, and ‘Oxidative stress and inflammation in psoriasis’, respectively. In the final section, ‘Oxidative stress and systemic cardiometabolic comorbidities in psoriasis’ additionally found literature is discussed.

### Oxidative stress and vascular alterations in psoriasis

Since the 1920s, histopathologists have described aberrant vascularization in psoriatic skin as follows: numerous dilated, tortuous and thin-walled capillaries (containing erythrocytes, but lacking pericytes or surrounding smooth muscle cells) in the upper part of the dermis in psoriatic lesions.^1,6,16–18^ In early phases of psoriatic plaque development, these capillaries already have augmented alkaline phosphatase activity and an increased flow prior to the occurrence of the keratinocyte hyperproliferation.^19–21^ Therefore, angiogenesis in psoriasis appears to be relevant to the development of a psoriatic lesion. Transformation of the superficial microvasculature during psoriatic evolution results in an angiogenic phenotype, generating the formation of the psoriatic lesion.

As angiogenesis is tightly regulated by a balance between pro- and antiangiogenic factors, the expression of proangiogenic factors must predominate the antiangiogenic stimuli in order for angiogenesis to occur.

The proangiogenic factors, hypoxia-inducible factor (HIF)-1α and HIF-2α, are significantly increased within the psoriatic plaque.^21–26^ In addition, HIF-1α is also increased in uninvolved psoriatic skin,^21,25^ which lends support to a relevant role in the pathogenesis of psoriasis. There appears to be a significant difference in the pattern of HIF-1α expression between normal skin and clinically involved, as well as uninvolved psoriatic cases;^21,25^ in psoriatic skin a nucleo­cytoplasmic pattern has been found, while in normal skin HIF expression appears to be only localized in the cytoplasm.

Low oxygen tension – hypoxia – is a main inducer of angiogenesis.^27–30^ HIFs, heterodimeric transcription factors composed of a regulatory oxygen-sensitive α-subunit (HIF-1α, HIF-2α and HIF-3α) and a constitutively expressed ­β-subunit (aryl hydrocarbon receptor nuclear translocators: ARNT, ARNT2, ARNTL),^13,31–35^ initiate the metabolic response to decreased oxygen tension. HIF-1 is expressed and detected in immune cell populations, including macrophages, neutrophils and dendritic cells, as well as in T and B lymphocytes and immune lymphoid cells (ILC1, ILC2, ILC3).^25,36^

HIF-2 expression is predominantly in the heart, lung, kidney and placenta in a range of cell types, including endothelial cells and certain immune cells (e.g. myeloid cells and lymphoid cells). Little is known about HIF-3, which is expressed mainly by epithelial cells in the lung and kidney.^36,37^ At physiological oxygen pressure, the HIF-α subunits are continuedly synthesized and degraded by the proteasome system. At normal oxygen concentrations, the α-subunit of HIF is hydroxylated by prolyl hydroxylation, allowing their recognition by Von Hippel–Lindau (VHL) tumour suppressor protein and proteasomal degradation. In hypoxic conditions, prolyl hydroxylases are inactive, resulting in absence of HIF-α degradation and subsequently causing increasing HIF concentrations.^13,38^

HIF expression appears most pronounced in the perivascular area; the area where one would least expect hypoxia. Then again, as oxygen saturation is a balance between supply and consumption, the oxygen consumption (e.g. by endothelial cells, inflammatory cells and stromal cells) might exceed the oxygen supply, leading to relative hypoxia.^22^ HIF-1α regulates vascular endothelial growth factor (VEGF) and its receptor Flt-1, which play a pivotal role in psoriatic angiogenesis,^39–41^ while HIF-2α and angiopoietin increase the capillary permeability. Therefore, expression of HIF-1α corresponds strongly with VEGF and endothelial cell proliferation.^21,23,42,43^ In addition, Tovar-Castillo *et al*. observed suppressed VHL mRNA and protein, and increased VEGF expression in psoriatic skin, while the expression of VHL was high and VEGF was low in healthy control participants. The results suggest that suppressed VHL levels fail to downregulate the expression of HIF and subsequently VEGF concentrations in psoriatic skin, which converts the proangiogenic phenotype seen in psoriasis.^44^

In accordance with this, Li *et al*. demonstrated that the microRNA miR-150 effectively regulates HIF-1α and VEGF-A expression and inhibits the stimulating effects of HIF-1 α and VEGF-A on angiogenesis and proliferation of keratinocytes by directly binding to their 3′ untranslated region receptor.^26^ A strong inverse correlation was found between down­regulation of miR-150 and upregulation of HIF-1α, and the severity of psoriasis (in terms of disease duration, severity index and body surface area involved). These changes can also contribute to the proangiogenic climate found in psoriasis pathogenesis.^26,45^

Short-time cutaneous expression of VEGF does not produce psoriatic lesions.^39,46^ However, data suggest that long-term VEGF activation in the cutis is necessary and sufficient to induce psoriatic formation, as opposed to inflammatory cytokines [including tumour necrosis factor (TNF)-α and interleukin (IL)-17] and keratinocyte growth factors which are not able to induce the complete psoriatic phenotype by themselves.^45,47–50^

Theoretically, HIF activation in T cells could contribute to oxidative stress and hypoxia in the pathophysiology of psoriasis, given the fact that HIF is also involved in T-cell survival and function. However, Rosenberger *et al*. deemed most of the CD8 T lymphocytes in established psoriatic plaques HIF-negative.^22^

Interestingly, the genetics of psoriasis also lends support to a crucial role of oxidative stress in the pathogenesis of psoriasis. Genetic polymorphism in glutathione S-transferase genes, coding for antioxidant enzymes, normally protecting against oxidative stress, inflammation and genotoxicity, appear to be strongly associated with an increased risk of developing psoriasis.^51–53^

### Oxidative stress and inflammation in psoriasis

Over the past half century, our understanding of the immunopathogenesis of psoriasis has evolved into a branching model of innate and acquired immunity. Epidermal challenge and presentation of autoantigens may play an initiating role. Dendritic cells may activate T-helper (Th) 17, Th22 and Th1c, generating IL-17A and IL-17F as key cytokines. IL-17A and IL-17F stimulate epidermal cells to express cytokines and chemokines, activating epidermal cells and endothelial cells, and attract various immunocytes including neutrophils.^1,54,55^ Epidermal cells express host defence proteins, including LL-37, which subsequently induces interferon (IFN)-α responses in plasmacytoid dendritic cells. In many segments of the immunopathogenesis TNF-α is a key cytokine.^1,6,54,56,57^

So far, the inflammatory components mentioned above in the immunopathogenesis of psoriasis may have cross-talk with oxidative stress, ROS, nicotinamide adenine dinucleotide phosphate (NADPH) oxidase activity (a major source of ROS) and HIF-1α/2α (an enzyme essential in regulating HIF-1 activity). As stated before, oxidative stress is significantly increased in (uninvolved) psoriatic skin (elevated HIF-1α levels).^21–24,26,58^

Whether a genetic predisposition or dysfunctional antioxidant defence system primarily causes oxidative stress (and secondary inflammation) and/or hypoxia is the result of developed inflammation has not yet been elucidated.

What has been shown is that polymorphisms in the glutathione S-transferase M1/T1 gene and/or the psoriasis susceptibility locus (PSORS1) cause a significantly higher prevalence of the development of psoriasis.^51,52,59^ The glutathione S-transferase M1/T1 gene is a genetic code responsible for a family of enzymes involved in the second line of defence against oxidative stress, while several genes in the PSORS1 locus are involved in the major histocompatibility complex, and therefore in antigen presentation to CD8^+^ T lymphocytes and subsequent cytokine production (eventually leading to oxidative stress).^59^

An argument for a role of oxidative stress in the pathogenesis of psoriasis is the fact that tobacco and (excessive) alcohol use, both recognized as major risk factors for developing and worsening of psoriasis, are known to cause oxidative stress and inflammation.^1,60–62^ These same factors are closely linked to the induction of oxidative stress and subsequent cardiovascular disease as well.^63–70^

HIF has already been studied in relation to psoriasis in greater detail. In physiologically hypoxic conditions, HIFs contribute to innate and adaptive immune cell homeostasis, whereas in morbid hypoxia, HIF signalling could in fact trigger tissue damage and immune cell dysfunction. HIF can alter immune cell function in various different metabolic pathways, including glycolysis, fatty acid synthesis, the tricarboxylic acid cycle, the pentose phosphate pathway or amino acid metabolism.^71^ Through these pathways, HIF appears to be able to exert influencing and regulatory properties on virtually all essential innate immune cells [e.g. mast cells, macrophages, (immature) dendritic cells and neutrophils] and adaptive immune cells (including T lymphocytes) involved in the pathogenesis of psoriasis (see Table 1).

**Table 1:** The hypoxia inducible factor (HIF)-1α-mediated effects on different cell types

Cell type	HIF-1α-mediated effects
Neutrophils	Increasing bactericidal activity
	Stimulating migration, invasion and survival of neutrophils
	Production of proinflammatory cytokines and nitric oxide
Basophils, eosinophils and mast cells	Stimulates survival, function and chemotaxis
	Stimulation of production of IL-6, IL-8, TNF-α and VEGF
Macrophages (classic M1, regulatory M2)	Increasing motility, invasion, phagocytosis, bactericidal activity and tumorigenic potential
	Chemotaxis
	Increasing secretion of proinflammatory cytokines and TLR-4 expression
Dendritic cells (DCs)	Stimulates survival and migration of DCs
	Secretion of proinflammatory cytokines (IFN-γ, IL-10, IL-22)
	Stimulates production, differentiation, activation and antigen presentation of DCs
	Stimulates T-cell activation
T lymphocytes (T-helper CD4^+^ T cells, cytotoxic CD8^+^ T cells)	Increase survival, proliferation, Th17 and Treg differentiation, migration and metabolic reprogramming of T cells
	CD4^+^ T cells: increased IL-17A production
	CD8^+^ T cells: increased cytolytic activity, granzyme and perforin production and expression of costimulatory/inhibitory molecules (CTLA-4, GITR, 4-1BB)
B lymphocytes	Stimulates (abnormal) B-cell development and autoimmunity, proliferation and prolonged survival

CTLA-4, cytotoxic T-lymphocyte-associated protein 4; GITR, glucocorticoid-induced (TNF superfamily related) protein; IFN-γ, interferon-γ; IL, interleukin; TNF-α, tumour necrosis factor-α; TLR, Toll-like receptor; Th17, T-helper cell 17; Treg, regulatory T cell; VEGF, vascular endothelial growth factor; 4-1BB (CD137), a member of the TNF receptor superfamily T-cell costimulatory receptor.^72,73^

HIF stimulates survival, migration and function in all innate immune cells such as mast cells, macrophages, neutrophils, natural killer cells and dendritic cells. Activity of these innate immune cells subsequently contributes to the development of oxidative and proteolytic stress, thereby enhancing the psoriatic inflammation and stimulating HIF production, all of which causes a vicious circle.

As stress of any kind, as well as skin conditions, may cause enhancement of psoriasis, an insufficiency of the antioxidant defence and the antiprotease system facing the enhanced release of innate immune cells with the incapacity to prevent HIF accumulation might result in an uncontrolled inflammatory process.^74–76^

HIF stimulation on innate immune cells appears to stimulate essential cytokines, chemokines and growth factors in the genesis of psoriasis; HIF stimulation on neutrophils activates the secretion of, for example, IL-12 and IL-17, while mast cell stimulation has a big impact on the expression of IL-6, CXCLs (chemokines) and VEGF levels.^13,14,29,76–79^

Dendritic cells serve as a bridge between innate and adaptive immunity and are masters in presenting phagocytosed antigens to T cells, and therefore they are vitally important cells controlling immunity and enforcing tolerance to self-antigens. HIF signalling affects dendritic cell functions, including survival, differentiation, maturation, migration and antigen presentation. Dendritic cells produce TNF-α, IFN-γ, IL-6, IL-12, IL-22 and IL-23, of which IL-22 and IFN-γ, in particular, appear to be regulated by HIF.^13,14,34,54,77,79–83^

Stimulation of CD4^+^ T cells of the adaptive immune system with specific antigen and cytokine signalling present in the microenvironment causes cell differentiation into the different T-cell subpopulations: Th1, Th2, Th17 or regulatory T cells (Tregs). Th1 cells require IFN-γ and IL-12 for their differentiation, while Th17 cells are induced by IL-6 and transforming growth factor-β in a HIF-1α-dependent manner; IL-23 subsequently promotes the expansion of Th17 cells.

Several studies have described a strong association between HIF-1α and IL-6 levels in psoriasis,^80,81^ although it is important to consider that HIF-1α and IL-6 expression both increase under hypoxic conditions.^84^ Some intimate that IL-6 and HIF-1α are closely related, as IL-6 may trigger a signalling cascade involving activation of signal transducer and activator of transcription (STAT) 3, which may further increase the expression of HIF-1α.^80^ Moreover, IL-6 may stimulate the expression of HIF-1α in Th17 cells in a STAT3-dependent manner. However, so far, there is no convincing evidence of the therapeutic efficiency of anti-IL-6 inhibition in psoriasis treatment.

The retinoic acid-related orphan receptor γt (RORγt) is the key transcription factor that drives Th17 differentiation. Mounting evidence is available that activation of RORγt and, subsequently, Th17 development is regulated through a mammalian target of rapamycin (mTOR)–HIF-1α axis. Augmented expression of HIF-1α might enhance the maturation of Th17 cells and attenuate the production of Tregs. This results in an increasing Th17 and Treg cell ratio, and a reduction in Treg production,^54,78^ causing restraint of the proliferation of pathogenic T cells.^85^ Of note, HIF-1α also tips the balance between Th17 and Treg differentiation, and indirectly promotes autoimmunity in patients.

Th1 cells secrete the chemokines IFN-γ and TNF-α, while pro­inflammatory cytokines such as IL-17 and IL-22 are derived from Th17 cells. IL-23 stimulates expansion of Th17 cells and can stimulate gamma delta T cells (γδT cells) to produce IL-17 in the absence of Th17 cells. Comprehensive research has revealed the essential role of cytokines, including IL-17, IL-23 and TNF-α, in the genesis of psoriasis.^1,6,54,55,86–89^ Exploration has demonstrated that IL-17 and HIF-1α levels are linked; augmented IL-17 expression is associated with increased HIF-1α, while downregulation of HIF-1α has been linked to lower IL-17 production.^90,91^ Some studies document a possible association between HIF-1α and IL-23, both promoting the Th17 and Th1 cell differentiation through targeting STAT6 and LYN (Lck/Yes Novel) tyrosine kinase, thereby causing the immune and inflammatory response in the peripheral blood and skin lesions of psoriasis.^79,92^ A close relationship is also reported between TNF-α and HIF-1α, where TNF-α production appears to be regulated by HIF-1α.^56,79^ These data suggest that HIF interferes with crucial inflammatory pathways in the pathogenesis of psoriasis.

HIF activation in inflammatory cells could contribute to oxidative stress and hypoxia in psoriasis development, given the fact that HIF is involved in T-cell survival and function. The more central in the psoriatic plaque, which is characterized by extreme influx and presence of inflammatory cells, the more extensive the hypoxia. However, research has established evidence that CD8 T lymphocytes in psoriatic plaques are HIF-negative,^22^ and proof has been found for extrinsic and intrinsic oxidative stress in psoriatic fibroblasts in the absence of T-lymphocyte infiltrates.^22,93^

Accordingly, in the psoriatic plaque, extracellular ROS overproduction by fibroblasts can exert a proinflammatory role in psoriatic skin independently and in concomitance with infiltrating T cells. Therefore, increased ROS along with insufficient total antioxidant capacity, as well as apoptosis via mitochondria, may be involved in the pathogenesis of psoriasis.^81^

### Oxidative stress and systemic cardiometabolic comorbidities in psoriasis

Oxidative stress is believed to be the cause of ageing and cardiovascular diseases. Cardiovascular diseases are complex entities with heterogenous pathophysiological mechanisms in which increased oxidative stress has been viewed as one of the potential common aetiologies. A variety of cardiovascular diseases, including atherosclerosis, heart failure and cardiac arrhythmias, have been shown to be associated with an excess of ROS.^68–70,94,95^ In addition, conditions predisposing to atherosclerosis such as diabetes, hypercholesterolaemia, hypertension and smoking are also associated with increased production of ROS.^62–65,67,68,96–101^

In physiology, a fine balance between the presence of ROS and antioxidants is essential for proper normal functioning of the cell. A basal level of ROS is indispensable for the manifestation of cellular functions, while excessive ROS levels result in damage to cellular macromolecules such as DNA, lipids and proteins, eventually causing necrosis and apoptotic cell death.^102^

Oxidative stress, characterized by an increased production of free oxygen radicals, represents a pivotal factor in the pathogenetic process of atherosclerosis by causing endothelial dysfunction with a vascular inflammatory response and oxidative modification of low-density lipoproteins (LDLs) among others. An increased ROS level causes endothelial dysfunction, resulting in decreased nitric oxide availability and vasoconstriction (as a result of angiotensin II stimulation), which both promote arterial hypertension. Moreover, ROS damage erythrocytes and activate white blood cells, which results in decreased (damaged) erythrocytes, increased reticulocytes and active leucocytes. As a consequence, blood viscosity and blood flow (in the affected area) decrease, and hypoxia and inflammation are further enhanced. In addition, ROS negatively influence myocardial calcium handling, from which arrhythmia and hypertrophic cardiomyopathy derive as a consequence of disbalanced cardiac remodelling due to hypertrophic signalling and apoptosis.^97,103^

In atherosclerosis, the leading cause of cardiovascular death in developed countries, oxidative stress fulfills an essential part in the atheroma formation next to activation of proinflammatory signalling pathways and expression of cytokine/chemokines. ‘Delinquent’ stimuli (i.e. dyslipidaemia, hypertension, diabetes, obesity and cigarette smoking) cause inflammation and oxidative stress, achieving qualitative changes in endothelial cells.

Subsequently, these changes generate endothelial cell dysfunction, which breaches the balance between vasodilatation and vasoconstriction, augments endothelial permeability and triggers the expression of adhesion and chemotactic molecules on the endothelial cell. As a result, inflammatory cells from the circulation are able to infiltrate the vessel wall.^104,105^ This facilitates the entry of LDL particles into the atrial wall and subsequent retention as a result of the binding of apolipoprotein B100 to proteoglycans of the extracellular matrix.

However, LDL needs to undergo numerous chemical modifications in the blood plasma before it becomes ‘atherogenic’ LDL.^104,106,107^ Augmented oxidative stress and related superoxide anion formation in vascular cells promote the conversion of LDL to a more atherogenic oxidized LDL (oxLDL). OxLDL particles stimulate the release of bioactive phospholipids capable of activating the endothelial cells to express various types of leucocyte adhesion molecules, including, for example, vascular cell adhesion molecule 1 (VCAM-1) and P- and E-selectins, which mediate rolling, adhesion and infiltration of circulatory leucocytes (monocytes and T cells) on the vascular wall and into the subendothelial space of the arterial wall. Once inside the intima layer, monocytes differentiate into macrophages which internalize modified lipoproteins (oxLDL), incorporate apoptotic cell fragments and bacterial endotoxins, causing lipid accumulation and foam cells formation, and eventually resulting in an atheromatous plaque.^104,106–109^

Extensive research has demonstrated an increased level of total oxidative stress in psoriasis and underlines the importance of oxidative stress in the development of psoriasis.^21–26^ Therefore, research into oxidative stress in psoriasis is becoming increasingly important, not only to further the knowledge of the role of oxidative stress in the pathogenesis of psoriasis itself, but also to improve insight into the many potential comorbidities that can occur in psoriasis.

In recent years, there has been mounting evidence that psoriasis does not solely affect the skin and joints, but is a systemic inflammatory disease associated with an increased prevalence of metabolic disorders (i.e. hypertension, diabetes, dyslipidaemia and subclinical atherosclerosis) and elevated risk of development of severe cardiovascular and cerebrovascular events.^9,110–117^ Previous studies have proven that mortality rates are increased in patients with psoriasis compared with healthy controls and that the life expectancy of patients with moderate-to-severe psoriasis is reduced by approximately 5 years, mainly due to cardiovascular comorbidities.^118–121^ In addition, a greatly increased economic and healthcare burden has been found to be associated with the presence of cardiovascular morbidities in individuals with psoriasis.^122–124^

Moreover, a correlation has been found between cardiac disease (including myocardial infarction and cardiac mortality) and cerebrovascular disease (stroke) and the severity of psoriasis.^114,115,125–133^ The relative risk of cardiovascular disease is greatest in younger patients, but is still significantly augmented in older patients aged ≥60 years. Research has shown that psoriasis is an important risk factor for ischaemic heart disease and atherosclerosis. Patients with psoriasis have increased arterial stiffness compared with healthy volunteers, and there is a positive relationship between arterial stiffness and psoriasis disease duration.^134–137^ Moreover, a reduction in psoriasis disease severity has been linked to an improvement in coronary atherosclerosis and the improvement of aspects of metabolic syndrome.^116,138–143^

An increasing number of studies are demonstrating a significantly higher prevalence of dyslipidaemia, decreased high-density lipoprotein levels and an increased LDL level in patients with psoriasis. In addition, the susceptibility of LDL to oxidation and increased formation of atherogenic oxLDL is increased in patients with psoriasis.^96,140,144–155^ Apart from blood cholesterol abnormalities, serum triglyceride levels are increased in patients with psoriasis compared with healthy controls.^144,147,149,156–159^ In addition, there appears to be a temporal correlation between hypercholesterolaemia and the development of psoriasis. For example, the Nurses’ Health Study II revealed an increased risk of developing psoriasis in patients with hypercholesterolaemia (hazard ratio 1.25) and the risk is augmented for patients with a longer duration of hypercholesterolaemia (especially >7 years).^160^

Unfortunately, the exact relationship between cardiovascular disease and psoriasis has not yet been elucidated, but these diseases appear to be intertwined in their pathomechanisms. For instance, it has been shown that people with obesity, diabetes mellitus and/or hypertension have a higher risk of developing psoriasis.^161–164^ Interestingly, the treatment of patients with psoriasis with metabolic disorders with metformin has been demonstrated not only to improve parameters of the metabolic syndrome, but also to decrease the severity of psoriasis.^165–167^ In addition, it has been shown that the prescription of metformin and thiazolidinediones to patients with diabetes reduces the risk of psoriasis development.^168–170^

Furthermore, with the treatment and improvement of psoriasis, oxidative stress and metabolic complications also appear to improve.^171–173^ There is growing evidence that oxidative stress plays a significant role and is prominently involved in psoriasis, as well as in metabolic syndrome and its related individual conditions (diabetes, hypertension, hypercholesterolaemia and/or atherosclerosis).^64,174–177^

The question is whether oxidative stress and its consequences could be a common component in the pathogenesis of psoriasis and cardiovascular diseases. This hypothesis could be supported by the fact that several studies have found evidence that a mutation in the glutathione S-transferase M1/T1 gene, a gene responsible in the second line of defence against oxidative stress, is not only associated with a higher prevalence of psoriasis, but also with myocardial infarction, hypertension, atherosclerosis and/or diabetes.^51,52,178–182^ However, not all researchers were able to find a correlation between oxidative stress and metabolic syndrome.^183^

Vasculature, and in particular hypoxia, is an overlooked area in reviews of the pathogenesis of psoriasis. Many years ago, the squirting papillae were described within the histopathogenesis of psoriasis. However, although several groups have studied hypoxia in psoriasis, the connection between hypoxia and microvasculature and inflammation has not yet been made. This review presents arguments that underline the importance of hypoxia and oxidative stress in the inflammatory and vascular response within the pathogenesis of psoriasis and associated cardiovascular and various metabolic diseases. Psoriasis is not a disease only affecting the skin and joints, but rather a systemic auto­inflammatory disease that causes internal damage giving rise to the various metabolic and cardiovascular disorders. The presence of common oxidative stress (and secondary) inflammatory pathways could provide a new explanation for the association between psoriasis and metabolic/cardiovascular comorbidities.

Oxidative stress seems to be able to develop into psoriasis or one of the various (in)complete forms of the metabolic syndrome. Once one of these disorders is present, other diseases also induced by oxidative stress may arise. It is attractive to speculate that oxidative stress could be a ‘trait-d’union’ between psoriasis and cardiovascular disease under the umbrella of the concept of ‘psoriasis a systemic disease of inflammation’. Further studies delineating the association between oxidative stress in psoriasis and cardiovascular disease might shed new light on this intriguing relationship.

## Data Availability

Data files of the literature search and article assessment are stored at the Department of Dermatology server at the RadboudUMC (University Medical Centre). Hardcopy articles of the search are stored in cabinets within the Department of Dermatology at de RadboudUMC.
